# Longitudinal lactate levels from routine point-of-care monitoring in adult Malawian antiretroviral therapy patients: associations with stavudine toxicities

**DOI:** 10.1093/trstmh/trt074

**Published:** 2013-08-07

**Authors:** Newton Chagoma, Jane Mallewa, Symon Kaunda, Yasin Njalale, Elizabeth Kampira, Mavuto Mukaka, Robert S Heyderman, Joep J van Oosterhout

**Affiliations:** a Malawi-Liverpool Wellcome Trust Clinical Research Programme, University of Malawi College of Medicine; b Department of Medicine, University of Malawi College of Medicine; c Department of Pathology, University of Malawi College of Medicine; d Division of Human Genetics, Faculty of Health Sciences, University of Cape Town, South Africa; e Dignitas International, Zomba, Malawi

**Keywords:** Lactate, Monitoring, HIV, Antiretroviral therapy, Stavudine, Toxicity, Malawi

## Abstract

**Introduction:**

Stavudine is still widely used in under-resourced settings such as Malawi due to its low price. It frequently causes peripheral neuropathy and lipodystrophy and increases the risk of lactic acidosis and other high lactate syndromes.

**Methods:**

We studied the association of longitudinal lactate levels, obtained by routine, 3-monthly point-of-care monitoring, with peripheral neuropathy, lipodystrophy and high lactate syndromes in adult Malawians who were in the second year of stavudine containing antiretroviral therapy (ART).

**Results:**

Point-of-care lactate measurements were feasible in a busy urban ART clinic. Of 1170 lactate levels collected from 253 patients over the course of one year, 487 (41.8%) were elevated (>2.2mg/dl), 58 (5.0%) were highly elevated (>3.5mg/dl). At least one elevated lactate level occurred in 210 (83.0%) of patients and sustained hyperlactatemia in 65 (26.4%). In random effects analyses lipodystrophy and peripheral neuropathy were associated with higher lactate levels. Only five patients developed high lactate syndromes (one lactic acidosis) of whom no preceding lactate measurements were available because events had started before enrolment. Lactate levels significantly decreased over time and no high lactate syndromes were observed after the 15th month on ART.

**Conclusion:**

Lipodystrophy and peripheral neuropathy were associated with higher lactate levels. Lactate levels decreased over time, coinciding with absence of new high lactate syndromes after the 15th month on ART.

## Introduction

Nucleoside reverse transcriptase inhibitors cause mitochondrial toxicities,^1^ among which peripheral neuropathy and lipodystrophy are the most common and lactic acidosis is associated with a very high case-fatality rate. Routine lactate monitoring has been undertaken in several cohort studies in affluent settings with the aim of preventing lactic acidosis. However, elevated lactate levels had poor predictive value for the development of lactic acidosis, as increased levels of serum lactate were common and lactic acidosis very rare.^2–4^ Lipodystrophy and severe cases of peripheral neuropathy were associated with high lactate levels in some studies.^5–7^

Stavudine has a high propensity to cause mitochondrial toxicities, but is still widely used in first-line antiretroviral therapy (ART) in sub-Saharan Africa. Although WHO has recommended phasing out its use,^8^ this has not yet been possible due to financial constraints in Malawi, where around three-quarters of adults on ART were on a stavudine containing regimen at the end of 2012.^9^

Lactate assays have been logistically and technically demanding, preventing regular use in sub-Saharan Africa. More recently, inexpensive point-of-care, hand-held lactate analyzers have been introduced with encouraging results,^10,11^ but data on routine lactate monitoring are lacking from the region.

In a prospective cohort study of adult Malawians on stavudine based ART, we found that peripheral neuropathy and lipodystrophy were common after the first year on treatment and that incidence rates in the second year were also high. On the contrary, new cases of high lactate syndromes were uncommon after the first year on ART.^12^ Here we report on prospective, routine, point-of-care lactate monitoring in the same cohort with the objectives to study associations of major stavudine associated toxicities with lactate levels over time and to explore the clinical utility of routine lactate monitoring.

## Methods

### Patients and data collection

We did a prospective cohort study of HIV infected adults, aged 18 or older, who had just completed a year of stavudine containing ART in Blantyre, Malawi and continued on the same regimen. After enrolment we followed patients three-monthly and at intercurrent sick visits for one year and described stavudine associated toxicities comprehensively, as reported elsewhere.^12^ In brief, peripheral neuropathy diagnosis was based on characteristic symptoms that had started after ART initiation. We used the Lipodystrophy Case Definition Study-based questionnaire to diagnose lipodystrophy.^13^ The estimated creatinine clearance was determined at enrolment by the Cockroft-Gault method. Lactate was measured routinely at enrolment and at each follow-up visit with the hand-held Lactate Pro^®^ (Arkray Europe B.V., Amstelveen, the Netherlands) at the point of care. Finger prick capillary samples were taken, without the use of a tourniquet. Patients were rested and were checked for dehydration. If found dehydrated based on clinical signs, patients were rehydrated before a sample was taken. We did not routinely exclude malaria nor were patients routinely fasting. In patients with suspected high lactate syndromes, elevated values were confirmed with a repeat test plus determination of an anion gap and venous CO_2_ level at the Johns-Hopkins Project Laboratories Blantyre, subject to availability. The lactate level was considered elevated if >2.2 mmol/L and highly elevated if >3.5 mmol/L. Sustained hyperlactatemia was defined as having at least two consecutive elevated lactate levels plus at least 50% of the levels being elevated.^14^ We diagnosed three different high lactate syndromes: severe hyperlactatemia (lactate >5.0 mmol/L without symptoms); symptomatic hyperlactatemia (lactate >5.0 mmol/L and suggestive symptoms present); lactic acidosis (lactate >5.0 mmol/L, and suggestive symptoms present, and manifestations of metabolic acidosis, such as acidotic breathing and/or reduced venous CO_2_ [<21 mmol/L]). Suggestive symptoms were: nausea, vomiting, weakness, weight loss, progressive numbness of the legs, abdominal pain, and shortness of breath. Stavudine was stopped in all patients with high lactate syndromes and in severe cases of peripheral neuropathy or lipodystrophy.

### Ethical considerations

The study adhered to the principles of the Declaration of Helsinki and was approved by the College of Medicine Research and Ethics Committee of the University of Malawi College of Medicine (protocol number P.01/09/719). Written informed consent was obtained from all patients.

### Data analysis

Data were double entered and validated prior to statistical analysis with STATA software (version SE/11; 4905; STATA Corp., College Station, TX, USA). The lactate data were censored when a patient stopped stavudine. In the analyses, the peripheral neuropathy and lipodystrophy status were assigned irrespective of the time of diagnosis between enrolment and the end of follow-up. We used this approach because of the very gradual development of peripheral neuropathy and lipodystrophy and because we considered that elevated lactate levels could be a reflection of a common underlying pathophysiology, which is present as long as the patient is exposed to stavudine and in some cases might only appear after the diagnosis of peripheral neuropathy and/or lipodystrophy is made.

The probability of experiencing elevated and highly elevated lactate levels was estimated using Kaplan-Meier survival analysis. Patients with elevated or highly elevated lactate levels at enrolment were excluded from these analyses. Comparisons between patients with and without peripheral neuropathy and those with and without lipodystrophy were performed with log rank tests. Random effect regression models were used to assess factors associated with lactate levels. The Hausman and Breuschan-Pagan Lagrange Multiplier tests were decisive in using the random effect regression methods. The variables age, sex, body-mass index (BMI), estimated creatinine clearance, WHO stage at ART initiation, peripheral neuropathy and lipodystrophy were included in a univariate random effects regression model. Three random effects multivariable models were fitted subsequently with variables that had some degree of association in the univariate model (p < 0.1). One model also included a diagnosis of peripheral neuropathy as variable, a second included a diagnosis of lipodystrophy but not peripheral neuropathy and a third excluded both peripheral neuropathy and lipodystrophy. All tests were 2-sided and a p-value of < 0.05 was considered statistically significant.

## Results

We consecutively enrolled 253 patients between May and September 2009. There was a female preponderance 158/253 (62.5%) and the median age was 36 years (IQR 31–43). At the start of ART, the median CD4 count was 141 cells/µL and 166/244 (68.0%) had a CD4 count below 200 cells/µL; 106/253 (41.9%) were in WHO stage 3 or 4 at the start of ART. At enrolment the median BMI was 23.1 kg/m^2^ (IQR 21.2–25.5) and the median estimated creatinine clearance was 82.1 ml/min (IQR 70.1–98.7). After enrolment, i.e. during follow-up in the second year of ART, 1.2% of patients (3/253) died (none of the deaths were related to toxicity), 4.0% (10/253) defaulted, 1.2% (3/253) transferred to another clinic, one withdrew from the study; none stopped ART. Lipodystrophy was diagnosed in 37/253 (14.6%), of whom 9 (24.3%) had lipo-hypertrophy only, 13 (35.1%) had lipo-atrophy only, 11 (29.7%) had both and in 4 (10.8%) it was not recorded what type of lipodystrophy was present.

Of the 253 patients 89 (35.2%) had peripheral neuropathy. Fourteen patients switched from stavudine to zidovudine due to adverse effects^12^, following national guidelines. Among those were five with high lactate syndromes. In the other nine, mildly elevated lactate levels were present in four patients, while five had normal lactate levels at the time of stopping stavudine.

The total number of lactate measurements taken at enrolment and during follow-up was 1170; the number of measurements were five or more in 206 patients, four in 28 patients, three in 7 patients, two in 5 patients and 7 patients had one measurement while on stavudine. Out of all measurements, 489/1170 (41.8%) were elevated (>2.2mg/dl), and 58/1170 (5.0%) were highly elevated (>3.5mg/dl). Out of the 253 patients 210 (83.0%) had at least one elevated lactate level and 46 (18.2%) had at least one highly elevated level. Fifty-three patients had two elevated levels, 37 had three, 8 had four and 9 had five elevated levels. Sustained hyperlactatemia occurred in 64/246 (26.0%) patients. Using a more strict definition of sustained hyperlactatemia, with three instead of two consecutively elevated levels, 54/241 (22.4%) fulfilled the criteria.

**Figure 1.**
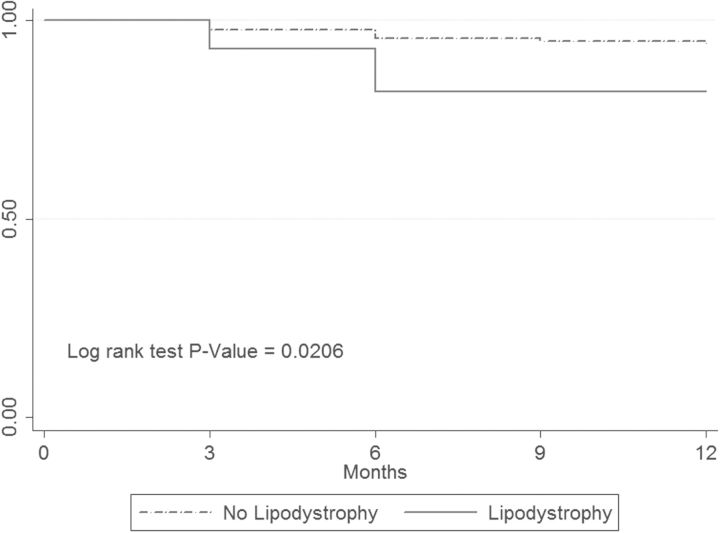
Time to highly elevated lactate level in patients with and without lipodystrophy diagnosis. The Y-axis indicates the fraction of patients who did not experience a highly elevated (>3.5 mmol/L) lactate level.

In Kaplan-Meier analyses, the probability of experiencing an elevated lactate level (>2.2 mmol/L) did not significantly differ between patients with and without peripheral neuropathy (log-rank p = 0.90) and between patients with and without lipodystrophy diagnoses (log-rank p = 0.54). The probability of a highly elevated lactate level (>3.5 mmol/L) did not differ between patients with and without peripheral neuropathy (log-rank p = 0.60) but was significantly higher in those with a lipodystrophy diagnosis (Figure 1).

In univariable random effects regression analyses, higher age, higher BMI, estimated creatinine clearance, calendar time on ART and diagnoses of peripheral neuropathy and lipodystrophy were associated with higher lactate levels, however in a multivariable model that excluded variables peripheral neuropathy and lipodystrophy, only age and calendar time on ART remained significantly associated. Lactate levels tended to get lower as time progressed. In further multivariable models, lipodystrophy diagnosis and peripheral neuropathy diagnoses were independently associated with higher lactate level (Table 1).

**Table 1.** Random effect regression analyses of factors associated with lactate levels during routine lactate monitoring

	Univariable analysis			Multivariable analysis^a^								
				Model 1			Model 2			Model 3		
	C	95% CI	p-value	C	95% CI	p-value	C	95% CI	p-value	C	95% CI	p-value
Age (years)	0.009	0.002 to 0.016	0.008	0.006	0.001 to 0.015	0.024	0.004	−0.001 to 0.009	NS	0.004	−0.001 to 0.010	NS
Sex	0.101	−0.025 to 0.226	NS	NA	NA	NA	NA	NA	NA	NA	NA	NA
BMI (kg/m^2^)	0.017	0.000 to 0.035	0.047	0.011	−0.007 to 0.030	NS	0.012	−0.019 to 0.026	NS	0.011	−0.002 to 0.026	NS
WHO-stage	0.053	−0.071 to 0.177	NS	NA	NA	NA	NA	NA	NA	NA	NA	NA
ECC (ml/min)	0.003	−0.000 to 0.006	0.061	0.001	−0.001 to 0.005	NS	0.001	−0.001 to 0.003	NS	0.001	−0.001 to 0.004	NS
Time (months)^b^	−0.204	−0.234 to −0.173	<0.001	−0.202	−0.231 to 0.171	<0.001	−0.201	−0.231 to −0.170	<0.001	−0.196	−0.227 to −0.166	<0.001
PN	0.149	0.022 to 0.277	0.021	NA	NA	NA	NA	NA	NA	0.113	0.011 to 0.255	0.03
LD	0.313	0.141 to 0.486	<0.001	NA	NA	NA	NA	NA	NA	0.192	0.049 to 0.334	0.008

BMI: body mass index; C: co-efficient; ECC: estimated creatinine clearance (Cockroft-Gault method); LD: lipodystrophy; NA: not applicable; NS: not significant; PN: peripheral neuropathy.

^a^Model 1 examines which factors are independently associated with lactate levels and excludes variables peripheral neuropathy and lipodystrophy. Models 2 and 3 examine whether peripheral neuropathy (model 2) and lipodystrophy (model 3) are independently associated with lactate levels.

^b^The variable time is the same as duration on stavudine containing ART.

During follow-up we diagnosed five cases of high lactate syndromes, all episodes were apparent before or at enrolment. Preceding lactate levels were not available in any of these cases, since all episodes had started before lactate monitoring was introduced. Three patients had symptomatic hyperlactatemia, one severe (asymptomatic) hyperlactatemia and one lactic acidosis; four were women, the median age was 36 years (range 29–52), the median BMI 25.5 kg/m^2^ (range 20.4–41.7), and the median lactate level at the time of presentation was 7.2 mmol/L (range 6.4–8.6). Three patients also had lipodystrophy and two had peripheral neuropathy. An anion gap and venous CO2 level was available from four out of the five patients. All had elevated anion gaps, but only the patient with lactic acidosis had a reduced CO2 level. All patients survived and restarted zidovudine-lamivudine-nevirapine without recurrence of a high lactate syndrome during follow up and lactate levels remained normal after the switch, except in one patient who had a raised level during a recurrent episode of dysentery. No high lactate syndromes were diagnosed beyond the 15th month after ART initiation.

## Discussion

We have described our experience with routine lactate monitoring and the pattern of longitudinal lactate levels in Malawian adults on first-line ART, information that was previously not available from African ART populations.

The point-of-care measurements were straight forward, quick to perform, easily learned by the nursing staff and therefore feasible in our circumstances. Because of the preparations and checks that needed to be done beforehand,^11^ the whole procedure was somewhat time-consuming. Routine lactate monitoring also had considerable costs, particularly due to the price of the test strips (more than US$2 each). However the availability of the hand-held analyzer at the clinic was a major improvement to the cumbersome procedure required for having venous samples transported and analyzed at the laboratory.

We first explored the association of lactate levels over time with routinely available patient characteristics in random effect regression analyses and found that higher age was independently associated with higher lactate levels, while time on ART was inversely associated with higher lactate levels. The association of age with higher lactate levels did not remain significant when either peripheral neuropathy or lipodystrophy diagnosis was introduced into the multivariable model, while both toxicities were independently associated with higher lactate levels. We had earlier described that higher age was an independent risk factor for the development of peripheral neuropathy and lipodystrophy in the same cohort.^12^ Taken together, these observations may suggest that elevated lactate levels reflect a common underlying pathophysiology of peripheral neuropathy and lipodystrophy that is exacerbated by higher age.

As lipodystrophy was most strongly associated with higher lactate levels, there may be a place for incorporating lactate level into a diagnostic algorithm of lipodystrophy for resource limited settings.^15^ This would add objectivity to a diagnostic tool that otherwise relies on patient's and clinician's observations only. The tool was derived from the original lipodystrophy case definition study that included anion gap rather than lactate level in its extensive and elaborate final diagnostic model.^14^ The authors admitted that anion gap is likely to be a surrogate for lactate level and that lactate level is probably a better marker for lipodystrophy, but they included anion gap because of the practical difficulties in measuring venous lactate. With the now available high-quality point-of-care tests this may be reconsidered.

The long-term metabolic consequences of having elevated lactate levels for individual patients remain unknown. Whether these individuals are at risk of adverse metabolic outcomes other than lipodystrophy needs to be determined.

We also found that lactate levels significantly decreased over time. Even so, elevated lactate levels were very common during routine lactate monitoring throughout the study period. The vast majority of patients experienced at least one elevated level, one quarter had sustained hyperlactatemia and nearly one fifth experienced at least one highly elevated level. Decreasing lactate levels coincided with a lack of new high lactate syndromes after the 15th month on ART. Of five patients with high lactate syndromes, four had accompanying suggestive symptoms. Because these episodes had started before routine lactate monitoring was introduced, we could not determine if they could have been identifiable earlier with routine lactate monitoring. None of the many other episodes of one or more elevated lactate levels proceeded to a high lactate syndrome, indicating that routine lactate monitoring is poorly predictive of high lactate syndromes after the first year on ART. This was further underlined by the fact that female gender and high BMI, characteristics that have consistently been observed as risk factors for high lactate syndromes, were not associated with elevated lactate levels.^16^ An important limitation of this study is that we did not measure lactate levels during the first year on stavudine based ART, when high lactate syndromes may be more prevalent.^16–18^ Until further studies have addressed this gap, final conclusions on the predictive value of routine monitoring and on its overall clinical utility, cannot be drawn. Availability of point-of-care lactate testing for patients with symptoms suggestive of symptomatic hyperlactatemia/lactic acidosis, a high index of suspicion by clinicians and adequate patient education may lead to reduced mortality from stavudine induced lactic acidosis due to earlier diagnosis and management. The replacement of stavudine with tenofovir in first line regimens in the region should contribute considerably to the primary prevention of high lactate syndromes and may even make targeted lactate measurements rarely needed.

### Conclusions

Point-of-care lactate measurements were feasible in a busy urban ART clinic in Malawi. Lactate levels obtained during routine lactate monitoring from adults in the second year of stavudine containing ART showed associations of lipodystrophy and peripheral neuropathy with higher lactate levels. Lactate levels decreased over time which coincided with a lack of new cases of high lactate syndromes after the 15th month on ART.
